# The prognostic value of peripheral blood parameters on all-frequency sudden sensorineural hearing loss

**DOI:** 10.1016/j.bjorl.2023.101302

**Published:** 2023-08-16

**Authors:** Hongcun Sun, Wenbo Jiang, Jian Wang

**Affiliations:** The Affiliated People’s Hospital of Ningbo University, Department of Otorhinolaryngology-Head and Neck Surgery, Ningbo, China

**Keywords:** Sudden hearing loss, Prognosis, Fibrinogen

## Abstract

**Objective:**

To determine whether peripheral blood parameters have any predictive value for all-frequency Sudden Sensorineural Hearing Loss (SSNHL).

**Methods:**

We chose 78 individuals with all-frequency SSNHL who had been admitted to our department. They were divided into two groups: the effective group and the ineffective group. In patients with all-frequency SSNHL, the prognostic variables, including peripheral blood tests and clinical traits, were examined by a logistic regression analysis. In addition, the predictive value was carried out.

**Results:**

The effective rate of all-frequency SSNHL was 61.5%. Pre-treatment hearing level and the proportion of patients with diabetes were both significantly lower in the effective group than in the ineffective group (*p* = 0.024 and 0.000, respectively). The levels of fibrinogen and C-reactive protein were also significantly different between the two groups (*p* = 0.001 and 0.025, respectively). Pre-treatment hearing level and fibrinogen level both significantly impacted the prognosis of all-frequency SSNHL (*p* = 0.032 and 0.002, respectively), according to a logistic regression analysis. Furthermore, the prognosis was significantly predicted by both fibrinogen level and pre-treatment hearing level (*p* = 0.001 and 0.0002, respectively). The receiver operating characteristic curve showed that the fibrinogen level had a sensitivity of 85.4% and a specificity of 60.0% for predicting the prognosis of all-frequency SSNHL.

**Conclusion:**

For the prognosis of all-frequency SSNHL, the fibrinogen level can be regarded as a useful predictor. The level of C-reactive protein, however, does not have a significant prognostic effect on predicting all-frequency SSNHL. Therefore, more attention should be devoted to the level of fibrinogen in the acute period of all-frequency SSNHL.

**Level of Evidence:**

4.

## Highlights

• The effective rate of all-frequency SSNHL is 61.5%.
• The fibrinogen level is a useful predictor for the prognosis of all-frequency SSNHL.
• CRP, NLR, and PLR don’t have a significant prognostic effect on all-frequency SSNHL.

## Introduction

Sudden Sensorineural Hearing Loss (SSNHL) is defined as sudden hearing loss of ≥30 dB over at least three continuous frequencies that develops within 72 h without any known cause.^1^ Actually, SSNHL is a frequent emergency.^2^ The incidence of SSNHL is thought to range from 5 to 27/100,000 people worldwide. Despite the absence of epidemiological data on SSNHL in China, clinical experience indicates that the condition appears to be on the rise and that more and more young people are developing it. Medical professionals should pay attention to the phenomenon. All-frequency SSNHL is characterized by a sudden hearing loss, tinnitus, and ear fullness, which frequently affects only one ear. These symptoms seriously impact study, work, and sleep. In general, the prognosis for all-frequency SSNHL is quite bad. Understanding the pertinent prognostic variables is essential.

The prognosis of SSNHL is influenced by numerous factors. The types of SSNHL have been shown to be a significant prognostic factor in earlier research. For instance, total-deafness SSNHL in four types has the worst prognosis. A retrospective investigation revealed that the prognosis of SSNHL was strongly correlated with age, vertigo, interval between onset and treatment initiation, and low-density lipoprotein levels.^3^ An earlier study found that the prognosis improved with younger age and earlier therapy.^4^ Additionally, the prognosis for those with profound hearing loss is bleak. In order to treat patients with severe SSNHL and alleviate their symptoms, clinicians must pay closer attention to these individuals.

The inflammatory response may be a crucial connection in these pathways and be linked to the severity of SSNHL, regardless of the pathophysiology, which may be an immunological response, a viral infection, or a microcirculation abnormality in the inner ear. As a result, in recent years, an increasing number of academics have started to concentrate on the connection between inflammation and SSNHL.

It is well known that pertinent peripheral blood measurements can frequently quantify an inflammatory response. Numerous studies have demonstrated that Platelet-Lymphocyte Ratio (PLR) and Neutrophil-Lymphocyte Ratio (NLR) levels can be utilized to assess inflammation. In addition, it has been established that there is a relationship between these inflammatory markers and the prognosis of SSNHL. According to data from 186 patients with SSNHL, NLR may be a useful prognostic factor for SSNHL, as the ability of hearing recovery decreased with the increase in NLR level.^5^ Based on one study, NLR may be regarded as a trustworthy indicator for doctors to predict the prognosis of SSNHL.^6^ The results of the other study, in which NLR and PLR levels of patients with SSNHL were significantly greater than those of healthy individuals, demonstrated that both NLR and PLR can be utilized as reliable predictive criteria for adolescent patients with SSNHL.^7^

Fibrinogen is a reliable biomarker as well as an important coagulation factor. It can be utilized as a crucial monitoring sign when all-frequency SSNHL is being treated. The fibrinogen level may therefore be crucial to the outcome of SSNHL. According to a meta-analysis research, there was no discernible change in fibrinogen level between SSNHL patients and the control group.^8^ As opposed to the no recovery group, the recovery group had a reduced fibrinogen level, which implies that a high fibrinogen level is linked to a bad prognosis for SSNHL. C-Reactive Protein (CRP) is a popular measure that reflects inflammation in the acute phase. It can be used to assess the prognosis of malignancies and inflammatory illnesses.^9^, ^10^ Contrarily, there is debate concerning the association between CRP level and the prognosis of all-frequency SSNHL.^11^

Therefore, the purpose of the current study is to determine whether certain peripheral blood parameters, such as NLR, PLR, CRP, and fibrinogen, are useful in predicting the prognosis of all-frequency SSNHL.

## Methods

### Patients

Patients with all-frequency SSNHL who were hospitalized in our department between January 2020 and March 2022 were included in this retrospective analysis. Pure-tone audiograms were used to define all-frequency SSNHL as sudden hearing loss at 0.25, 0.5, 1, 2, 4, and 8 KHz. The following requirements must be met by all patients: 1) Unilateral SSNHL; 2) Interval between onset and treatment initiation was ≤3 days; 3) No history of noise exposure, ototoxic drug application, Meniere's disease, ear trauma, and other inflammatory diseases in the affected ear; 4) No history of chronic inflammatory diseases, infectious diseases, chronic wasting diseases, as well as abnormal coagulation disorders. Furthermore, during the follow-up period, they were able to finish the treatment plan. After being admitted, patients were required to consent to Magnetic Resonance Imaging or Computed Tomography scan in order to rule out occupied lesions in the cerebellopontine angle area. These eligible patients were divided into two groups based on efficacy: the effective group and the ineffective group. The Ethical Committee of The Affiliated People’s Hospital of Ningbo University approved the collection of the data (2022-020).

### Hematological examinations

On the day of admission, routine blood tests, CRP tests, and fibrinogen tests were all completed prior to starting any treatment. Routine blood tests reveal the absolute amounts of neutrophils, lymphocytes, and platelets. There was a calculated NLR and PLR. The latex-enhanced immunoturbidimetry method and the CLAUSS method were used to measure the levels of CRP and fibrinogen, respectively. Following ranges were regarded as normal: CRP: 0–10 mg/L, fibrinogen: 2–4 g/L.

### Treatment option

Batroxobin was initially injected intravenously in a dose of 10 Bu once every other day for defibrinogenation therapy. The following dose, 5 BU, was administered a total of four times. Testing the amount of fibrinogen was important prior to the administration of batroxobin. Until the following day, batroxobin administration was prohibited if the fibrinogen level was less than 100 mg/dL. A methylprednisolone injection was also administered, starting at 80 mg per day for the first three days and then decreasing to 40 mg per day for the following three days. Intratympanic dexamethasone therapy was given a total of six times, once every two days. Through the anterior-inferior quadrant of the tympanic membrane, 0.5 mL of dexamethasone (4 mg/mL) was injected into the tympanic cavity as part of the therapy. Additionally, these patients agreed to receive hyperbaric oxygen therapy, which was given in a hyperbaric chamber once every day for a total of 20 days (2.0 atmospheric pressure). One month after the conclusion of the treatment option, pure-tone audiometry and acoustic immittance tests were conducted.

### Evaluation of hearing recovery

To evaluate hearing recovery, the average hearing threshold at 0.25, 0.5, 1, 2, 4, and 8 KHz was calculated. Hearing recovery was graded into two categories based on the typical increases in hearing following treatment.

Effective recovery: improvements in average hearing gains were ≥15 dB; average hearing returned to the normal level or to a level equal to the contralateral ear that was unaffected.

Ineffective recovery: improvements in average hearing gains were <15 dB.

### Statistical analysis

Data analysis was carried out using the Statistical Product and Service Solutions 19.0 software. The measurement data were presented in mean ± standard deviation (x ± s). Two independent samples were compared using a two-tailed, unpaired *t*-test. χ^2^ test was used to compare the rates. The prognostic factors were analyzed using logistic regression. The MedCalc software was used to create a Receiver Operating Characteristic (ROC) curve to assess the predictive ability. The statistical significance of the difference was shown by a *p*-value of less than 0.05.

## Results

In total, 78 eligible patients with all-frequency SSNHL were included in this study. Age, gender, affected side, vertigo, hyperlipidemia, diabetes, hypertension, pre-treatment hearing level, average hearing gains after treatment, and other common characteristics were gathered. Table 1 provides a summary of these patients’ profiles.

**Table 1:** Profiles of patients with all-frequency SSNHL.

Factors	Patients (n = 78)
Age (years)	54.1 ± 14.1
Male/female	33/45
Left/right ear	37/41
Vertigo	7
Hyperlipidemia	40
Diabetes	18
Hypertension	21
Pre-treatment hearing level (dB)	79.5 ± 17.8
Post-treatment hearing gains (dB)	23.5 ± 17.1
The effective number of patients	48

Table 2 shows that in the effective group, there were significantly fewer patients with diabetes and lower pre-treatment hearing levels than in the ineffective group (*p* = 0.024 and 0.000, respectively). The levels of fibrinogen and CRP were also significantly different between the two groups (*p* = 0.001 and 0.025, respectively). Other variables, however, did not significantly differ between the two groups (all *p* > 0.05).

**Table 2:** Comparison for related variables between two groups.

Factors	Effective group (n = 48)	Ineffective group (n = 30)	*t* (*χ^2^*)	*p*-value
Age (years)	54.2 ± 12.2	53.9 ± 16.7	0.093	0.926
Male/female	22/26	11/19	(0.416)	0.519
Left/right ear	23/25	14/16	(0.012)	0.914
Vertigo	4	3	(0.000)	1.000
Hyperlipidemia	24	16	(0.082)	0.744
Diabetes	7	11	(5.072)	0.024
Hypertension	14	7	(0.319)	0.572
Pre-treatment hearing level (dB)	73.4 ± 17.3	89.2 ± 19.8	3.710	0.000
Neutrophil counts (10^9^/L)	5.3 ± 2.8	6.0 ± 3.0	1.024	0.309
Lymphocyte counts (10^9^/L)	1.6 ± 0.5	1.5 ± 0.5	0.096	0.924
Platelet counts (10^9^/L)	234.1 ± 62.6	224.5 ± 48.2	0.723	0.472
NLR	4.0 ± 2.9	4.7 ± 3.5	0.917	0.362
PLR	178.6 ± 98.6	164.8 ± 73.4	0.660	0.512
CRP (mg/L)	2.5 ± 1.1	3.8 ± 3.7	2.279	0.025
Fibrinogen (g/L)	2.9 ± 0.4	3.2 ± 0.5	3.341	0.001

Understanding variables like CRP, fibrinogen, diabetes, and pre-treatment hearing level that were related to the prognosis of all-frequency SSNHL was done using multivariate logistic regression analysis. Table 3 demonstrates that pre-treatment hearing level and fibrinogen level were both significant variables that influenced the prognosis of all-frequency SSNHL (*p* = 0.032 and 0.002, respectively). Other variables did not change significantly (all *p* > 0.05).

**Table 3:** Multivariate analysis of prognostic factors in patients with all-frequency SSNHL.

Covariate	B value	*p*-value	Odds ratio	95% Confidence interval
CRP	−0.103	0.539	0.902	0.649‒1.253
Fibrinogen	−1.367	0.032	0.257	0.074‒0.893
Diabetes	−0.979	0.112	0.376	0.112‒1.257
Pre-treatment hearing level	−0.045	0.002	0.956	0.929‒0.984

**Figure 1:**
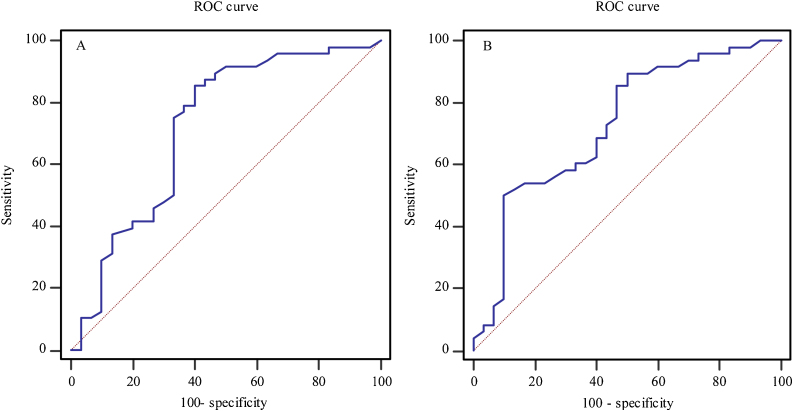
ROC curve of fibrinogen level (A) and pre-treatment hearing level (B) in patients with all-frequency SSNHL for predicting hearing recovery.

As shown in Fig. 1A, fibrinogen level showed a statistically significant prognostic value for hearing recovery in patients with all-frequency SSNHL (*p* = 0.0001). The area under the curve for fibrinogen level in predicting prognosis in individuals with all-frequency SSNHL, according to the ROC curve, was 0.720 (95% CI 0.607‒0.816). When the optima cutoff value was 3.19, the fibrinogen level had a sensitivity and specificity of 85.4% and 60.0%, respectively, in predicting the prognosis of all-frequency SSNHL.

Fig. 1B shows the prognosis of all-frequency SSNHL could also be predicted using pre-treatment hearing level (*p* = 0.0002). The area under the curve was 0.728 (95% CI 0.616‒0.823). With the optimal cutoff value (65.8), the predictive sensitivity and specificity were 50% and 90%, respectively.

## Discussion

The theory of inner ear ischemia infarction is still widely supported, despite the lack of recognized specific mechanisms for all-frequency SSNHL. All-frequency SSNHL is now treated with systemic glucocorticoid usage and defibrinogen therapy. The initial treatment option in this study included intratympanic steroid injection and hyperbaric oxygen therapy in addition to the drug therapy advised by the guidelines because it has been noted in numerous studies that this combination therapy may be extremely beneficial for hearing gains in all-frequency SSNHL.^12^, ^13^

All the participants in our study stuck it out until the completion of the follow-up. As a result, the effective rate of all-frequency SSNHL was 61.5%. Therefore, it is undeniable that the outlook is still dismal. Wen et al. reported that the more hearing loss at the initial onset, the worse the prognosis.^14^ Our study demonstrated that, when comparing associated characteristics between the effective and ineffective groups, diabetes and pre-treatment hearing level can have an impact on the prognosis of all-frequency SSNHL. Furthermore, pre-treatment hearing level was connected to the outcome of all-frequency SSNHL, according to multivariate analysis. It is inferred that the more hearing loss at the initial onset, the worse the prognosis of all-frequency SSNHL. However, for the prognosis of all-frequency SSNHL, diabetes had minimal predictive value. Nevertheless, it is crucial to monitor changes in blood glucose levels while receiving clinical treatment. In the meantime, this study has limitations. Because only a relatively small number of patients had vertigo, the impact of vertigo on hearing recovery was not identified.

Many years ago, some academics proposed the idea of “micro-inflammatory states”, which are frequently seen in ischemia and hypoxic disorders and are brought on by non-pathogenic microbial infection. In reality, the onset of a “micro-inflammatory state” frequently coincides with the release of inflammatory substances, which raises the relevant blood parameters. According to one study, the development of atherosclerosis may raise CRP levels, which in turn causes problems with lipid metabolism, platelet aggregation, and ultimately thrombosis.^15^ In a meta-analysis study, CRP levels were greater in SSNHL patients than in controls.^16^ Therefore, the connection between CRP level and the prognosis of all-frequency SSNHL deserves further research. The parameters NLR, PLR, and fibrinogen have also been mentioned by Doo et al. as being relevant for the prognosis of SSNHL.^17^

Blood tests such as neutrophil, lymphocyte, and platelet counts, as well as NLR, PLR, CRP, and fibrinogen, were used in our study to conduct a correlation analysis with the prognosis of all-frequency SSNHL. Our research has proven that these parameters, such as neutrophil, lymphocyte, and platelet counts, NLR, and PLR, are not related to the prognosis of all-frequency SSNHL in the acute period. Moreover, multivariate analysis revealed no association between CRP level and prognosis of all-frequency SSNHL, despite a large difference in CRP level between the effective and ineffective groups. It is implied that CRP cannot accurately reflect the “micro-inflammatory state” in all-frequency SSNHL and that additional useful indicators need to be researched because CRP level may be easily influenced by other factors in all-frequency SSNHL.

It was discovered, however, that fibrinogen level can influence the prognosis of all-frequency SSNHL. There have been numerous studies on the association between fibrinogen and SSMHL. Although one study found no link between high fibrinogen levels and circulating microparticles in SSNHL,^18^ subsequent researches demonstrated that fibrinogen level is an adverse factor in the prognosis of SSNHL.^19^, ^20^, ^21^ Our findings also demonstrate that fibrinogen level can affect the prognosis of all-frequency SSNHL. Thus, recent researches have confirmed that thrombosis in the inner ear may cause reduction in blood flow, which can result in oxygen deficiency and apoptosis of cochlear hair cells. As a result, thrombosis is the most probable cause of all-frequency SSNHL, and we should pay close attention to the coagulation status, particularly fibrinogen level. However, because the pathophysiology of low- and high-frequency SSNHL is not primarily caused by thrombosis, the role of fibrinogen is unclear, and its impact on prognosis is currently unappreciated. In general, it is important to pay closer attention to how the level of fibrinogen changes during treatment. This study investigated the predictive usefulness of fibrinogen level in more detail. The ROC curve revealed that fibrinogen level had a strong predictive value for all-frequency SSNHL, with a sensitivity of 85.4% and a specificity of 60.0%. Therefore, the fibrinogen level can be regarded as a useful predictor in predicting the prognosis of all-frequency SSNHL. More attention should be devoted to the level of fibrinogen in the acute period of all-frequency SSNHL. Early and standardized defibrinogen therapy is critical for improving efficacy.

In general, the findings of this study differed in part from those of earlier studies. The following factors were thought to be responsible: 1) Only all-frequency SSNHL was included in our study, whereas all types of SSNHL were included in the prior study; 2) Different factors were included in our study; 3) Significant differences in sample size; 4) Various laboratory testing techniques and treatment options; 5) Individual differences; and 6) Various criteria for evaluating the efficacy. Therefore, additional studies should be conducted to gradually comprehend these all-frequency SSNHL affecting elements.

## Conclusion

The prognosis for all-frequency SSNHL is shockingly bad. The important prognostic factors of all-frequency SSNHL were the main focus of this study. As a result, fibrinogen significantly altered the outcome for all-frequency SSNHL. Moreover, pre-treatment hearing level was a helpful and significant predictive predictor for all-frequency SSNHL. Then, with a sensitivity of 85.4% and a specificity of 60.0%, we discovered the value of fibrinogen level in predicting prognosis of all-frequency SSNHL. In brief, this study contends that the blood fibrinogen level is a reliable indicator of the prognosis for all-frequency SSNHL. CRP level, however, does not have a significant predictive effect on the prognosis of all-frequency SSNHL. Therefore, more attention should be devoted to the level of fibrinogen in the acute period of all-frequency SSNHL.

## Conflicts of interest

The authors declare no conflicts of interest.
